# Age-related alveolar bone maladaptation in adult orthodontics: finding new ways out

**DOI:** 10.1038/s41368-024-00319-7

**Published:** 2024-08-01

**Authors:** Yunfan Zhang, Jiale Yan, Yuning Zhang, Hao Liu, Bing Han, Weiran Li

**Affiliations:** Department of Orthodontics, Peking University School and Hospital of Stomatology & National Center for Stomatology & National Clinical Research Center for Oral Diseases & National Engineering Laboratory for Digital and Material Technology of Stomatology & Beijing Key Laboratory of Digital Stomatology & Research Center of Engineering and Technology for Computerized Dentistry Ministry of Health & NMPA Key Laboratory for Dental Materials, Beijing, China

**Keywords:** Ageing, Bone

## Abstract

Compared with teenage patients, adult patients generally show a slower rate of tooth movement and more pronounced alveolar bone loss during orthodontic treatment, indicating the maladaptation of alveolar bone homeostasis under orthodontic force. However, this phenomenon is not well-elucidated to date, leading to increased treatment difficulties and unsatisfactory treatment outcomes in adult orthodontics. Aiming to provide a comprehensive knowledge and further inspire insightful understanding towards this issue, this review summarizes the current evidence and underlying mechanisms. The age-related abatements in mechanosensing and mechanotransduction in adult cells and periodontal tissue may contribute to retarded and unbalanced bone metabolism, thus hindering alveolar bone reconstruction during orthodontic treatment. To this end, periodontal surgery, physical and chemical cues are being developed to reactivate or rejuvenate the aging periodontium and restore the dynamic equilibrium of orthodontic-mediated alveolar bone metabolism. We anticipate that this review will present a general overview of the role that aging plays in orthodontic alveolar bone metabolism and shed new light on the prospective ways out of the impasse.

## Introduction

**Fig. 1:**
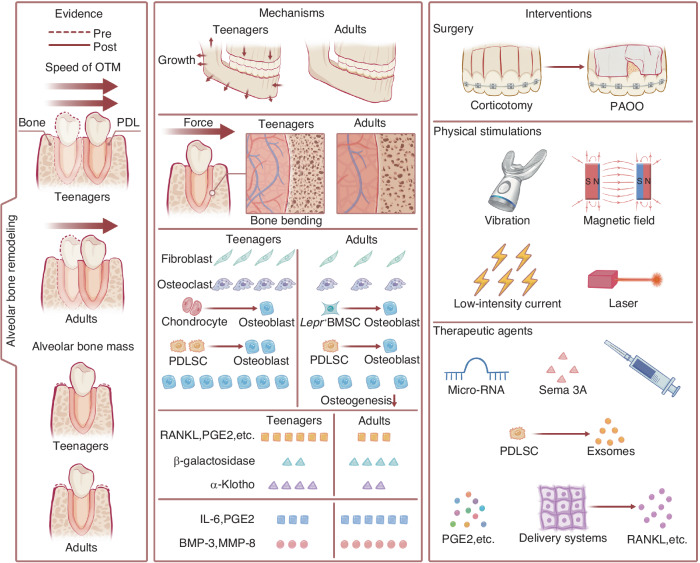
Graphic summary of the review. Three panels illustrate the age-related differences in orthodontic force-mediated alveolar bone remodeling, the underlying mechanisms, and possible interventions to address undesirable alveolar responses in adult orthodontics

Today, as clear aligner treatment flourishes worldwide and patients of all ages seek orthodontic treatment, the population of adults in the consulting room is ever-increasing.^1^ However, regardless of the type of appliance used to deliver orthodontic force, adults exhibit slower orthodontic tooth movement (OTM) and a greater incidence of open gingival embrasure and relatively noticeable alveolar bone loss,^2^ all of which can lead to dissatisfaction with the orthodontic experience and compromise health and aesthetics. However, although several hypotheses have been proposed, the etiology or underlying mechanisms still remain enigmatic. Since force-driven alveolar bone remodeling is the biological basis of orthodontics, the balance between bone resorption and bone formation determines the efficacy and outcome of orthodontic tooth movement. However, to the best of our knowledge, although alveolar bone is the most labile and active region of craniofacial bones, which possesses great plasticity to external mechanical stimuli,^3^ the stalled alveolar bone metabolism, or, the alveolar bone maladaptation to therapeutic loading occurs frequently in adults. As individuals move from their youth to senior years, the periodontium and the cells within inevitably undergo aging,^4–6^ affecting their microarchitecture and function, which could be the crux of this matter. From therapeutic perspective, several off-the-shelf or preclinical intervention strategies have been developed to shorten the total treatment time and preserve alveolar bone height and thickness in adult patients. We searched *PubMed*, *Web of Science,* and *Embase* with the terms as follows: (orthodontics [Mesh] OR orthodontic* OR tooth movement OR teeth movement), acceleration, (alveolar bone loss [Mesh]), (“age-related changes” OR aging), (“Periodontium”[Mesh] OR “Periodontal Ligament”[Mesh]), bone augmentation, (“gingival recession”) and their combination with “OR” or “AND”. This review consolidates the current understanding of age-related alterations in the maintenance of bone homeostasis and summarizes the existing evidence, possible underlying mechanisms and intervention programs for age-related differences in orthodontic-mediated alveolar remodeling that may improve understanding and provide new insights to address this issue (Fig. 1).

## Overview of skeletal metabolism: from infancy to old age

**Fig. 2:**
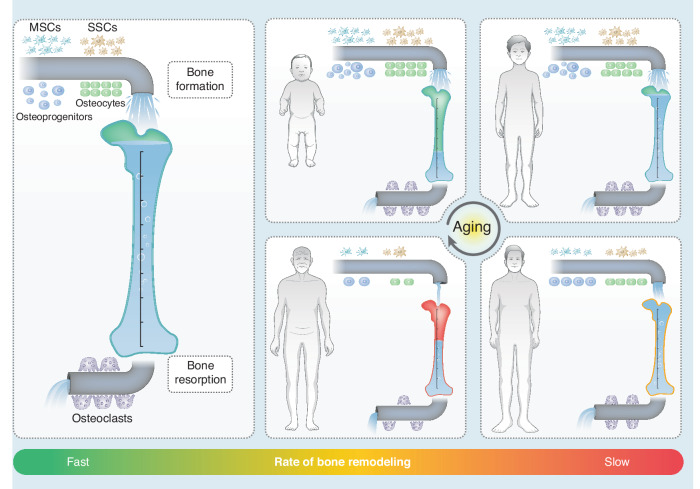
Graphic illustration of the trend of general skeletal metabolism with age

To better elucidate the underlying mechanisms of the histological and functional changes that take place in adult alveolar bone, herein, we first underscore the flow or trend of general skeletal metabolism with age (Fig. 2). The skeleton provides physical support, regulates calcium-phosphorous homeostasis and houses hematopoietic progenitors, which is an efficient and subtle “servo” system that renews and reconstructs in response to internal or external signals throughout the human full life cycle.^7^ Differentiated from mesenchymal stem cells (MSCs) or skeletal stem cells (SSCs), osteoblasts are the major source of bone matrix deposition and mineralization.^8^ Whereas osteoclasts dominate bone lysis or destruction. The ever-lasting “tug of war” determines all bone biological events, including development, defect regeneration and aging. From the embryonic phase through adolescence, bone anabolism and catabolism boost at several peaks of growth and development.^9^ Overall bone metabolism keeps at a high level due to the need for new bone morphogenesis and ossification. After reaching early adulthood, bone metabolism gradually slows down. Although no significant macroscopic changes in bone morphology occur, bone anabolism continues outstripping catabolism, leading to a sustained increase in bone volume and mineral density. After reaching a peak in bone mass at approximately 20–30 years of age, the counterbalance between bone formation and resorption is nip and tuck, maintaining bone mass and mechanical strength throughout adulthood.^8,10^ Irrespective of sex differences and age-related steroid hormone deficiency, as MSCs further pace to the senescence state, the altered proliferation and differentiation patterns and impaired functions of osteoprogenitors are responsible for the attenuation of osteogenesis, resulting in disturbed bone homeostasis and continued bone loss until reaching the diagnostic threshold, i.e., osteoporosis.^11,12^

Despite the different embryonal origins and ossification patterns, maxillofacial bones share the same metabolic trend of general skeleton.^13^ The velocity of bone remodeling and the changes in bone mass are distinct aspects depicting the process and the result of alveolar bone metabolism, respectively, both of which have important biological and clinical significance in orthodontics. Because of their different nature and clinical characteristics, two factors that reflect alveolar bone remodeling, OTM rate and alveolar bone mass, are dissect separately in this review.

## The OTM rate

When orthodontic force is applied, osteoclasts are activated at the compression sites, initiating bone resorption that can be observed and clinically evaluated as OTM. Hence, rate of OTM is a reliable indicator of remodeling efficiency and force-responsiveness of periodontium. Moreover, delayed OTM often leads to decreased patient compliance and increased morbidity from tooth demineralization and periodontal disease.^14^ However, few studies have been conducted to quantify the difference in OTM rates between adults and adolescents. Some retrospective studies reported that adult patients take 4 to 12 months longer to treat than adolescents.^2,15,16^ Zheng et al.^2^ demonstrated that the duration of correction for bimaxillary protrusion was 20.14 months and 24.91 months in adolescents and adults, respectively. Similarly, Furquim et al.^15^ reported that the average treatment time for Class II malocclusion was 3.32 years and 4.24 years in adolescents and adults, respectively. During a given period, the OTM distance was 1.4- to 1.6-fold greater in adolescents than in adults.^17–22^ A slower rate of tooth movement in adults has further been demonstrated in OTM rodent models. Both Ren et al.^23,24^ and Bridges et al.^25^ reported that the rate of tooth movement was the fastest during the first few weeks after orthodontic exposure and was approximately 2-fold greater in younger rats. Misawa et al.^26^ conducted standardized OTM assessments on rats in four age groups, 10-, 30-, 50-, and 80-week-old rats, and found that the amount of tooth movement in the 10-week-old group was significantly greater than that in the three other groups. Moreover, some studies did not find statistically significant differences, which may be ascribed to the limited sample size, use of an inappropriate age distribution, and poor oral hygiene.^27–29^ Although we already have a relatively clear clinical impression of this phenomenon, additional high-quality studies with a homogeneous design and larger sample sizes are encouraged to provide further validation.

### Underlying mechanisms

Activation of OTM requires pathways that translate mechanical stimuli into biological signals. Therefore, age-related alterations in the histology, cell biology, and microenvironment of the periodontium that may interfere with force perception, transmission and biological effects have been extensively investigated.

#### Histological and functional changes in periodontal tissue

Like other bones, alveolar bone density increases from puberty to adulthood due to continued mineral deposition. The density of normal alveolar bone increases with age from the teenage years through the third decade of life.^30,31^ Bridges et al.^25^ reported that the rate of tooth movement, including instantaneous movement, movement in the delay period, and late movement, was significantly lower in older rats than in younger rats at all three stages of OTM, i.e., instantaneous movement, delay period and late movement. By recording bone density during OTM, they found that the density of younger rats was lower than that of older rats at the corresponding stages. This evidence suggests that a higher density of alveolar bone may result in a greater workload for osteoclasts. This hypothesis could be supported to some extent by the fact that the rate of OTM in the maxilla was significantly higher than that in the mandible in the same subject, which was attributed to the difference in bone density.^27,32^ In addition, the rate of OTM was significantly higher in osteoporosis models than in controls. The negative correlation between OTM rate and trabecular bone volume was found to be statistically significant.^33^

**Fig. 3:**
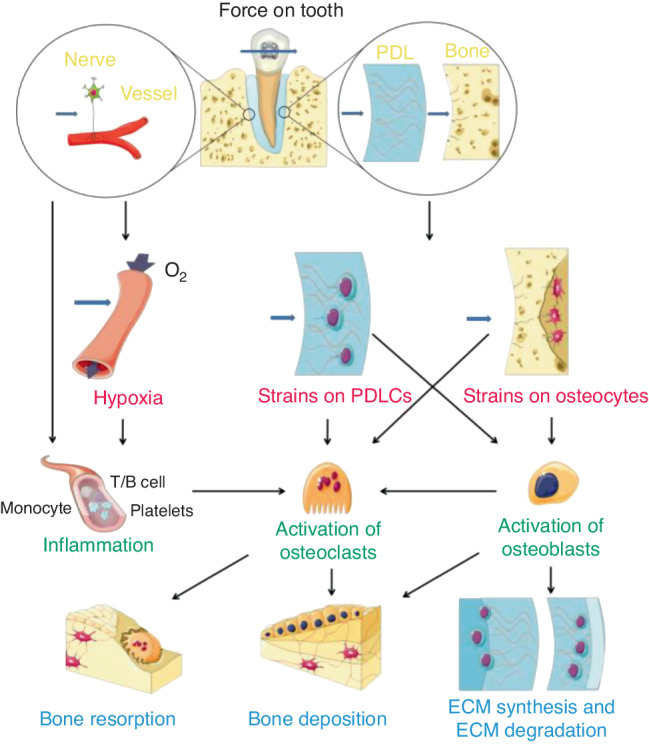
Schematic illustration of mechanosensing and transduction of orthodontic-mediated alveolar bone remodeling. Both PDLCs and osteocytes are important stress sensors and are important for the subsequent activation of osteoclasts. Open Access^35^ (from Li et al., *International Journal of Oral Science*, 2021, 13:20

When orthodontic force is applied, it is transmitted to all adjacent tissues. Orthodontic forces can cause matrix strain in the periodontal ligament (PDL) and alveolar bone, resulting in compression or stretching of collagen fibrils, altering the configuration of the extracellular matrix (ECM) and mechanical-sensitive cells, e.g., periodontal ligament cells (PDLCs), osteocytes, etc. Moreover, interstitial fluid flowing in the lacuna-tubule system can also generate shear stress on the surface of bone cells.^34^ These mechanical signals activate intracellular pathways and induce various cellular responses (Fig. 3).^35^ Age-related histologic changes in periodontal tissue may lead to a decrease in mechanical sensitivity and transduction. The PDL is the boundary of mechanosensing in the periodontium that initiates alveolar bone remodeling via orthodontic force.^36^ The distinct mechanical properties of the periodontium may result in the direct transmission of different forces. Age-related histologic changes have also been discovered in the PDL. A significant decrease in surface area, elasticity of the PDL, and the number of active osteoclasts, osteoblasts, fibroblasts, collagen fibers, and blood vessels was observed with age.^4,5,37–40^ The histological alterations observed in this study led to the conclusion that the reorganization of the PDL on the side experiencing pressure occurred earlier and more pronouncedly in young (6-week-old) rats than in old (9- to 12-month-old) rats.^37^ This suggests that the importance of age-related changes in the PDL structure in OTM. Similarly, the histological and mechanical properties of alveolar bone also change with age. In a study of 46 mice aged between 3 and 77 weeks revealed a decrease in femur bone density and the elastic modulus with age.^40^ In comparison to older mice, mice aged in excess of 12 weeks exhibited a reduction in the elastic modulus of approximately 10 GPa. It is evident that juvenile bones are more easily deformed than adult bones.^2^ A greater degree of deformation in a given area promotes rapid bone resorption^34^ and may contribute to the faster OTM observed in adolescents. Moreover, the decrease in the lacunar-canalicular volume with age may further contribute to the age-related increase in stiffness and brittleness of bone, which may lead to less matrix deformation and poorer cell mechanoperception.^31,41^

In addition to the age-related histologic changes that occur in periodontal tissue, the responsiveness of these force-sensitive cells might also be attenuated accordingly.^42^ After exposure to orthodontic force, the shape of PDLCs from younger donors exhibited more pronounced changes with more spindle-shaped irregularities and more surface extensions compared to those from adult donors, indicating better perception of mechanical perturbations. Furthermore, the expression of β-actin, a critical component of the cytoskeleton that is responsible for force transmission and response, was greater in PDLCs from younger donors both before and after exposure to orthodontic force. These findings may indicate that aging leads to a reduction in the activity and responsiveness of PDLCs.^42^ Apart from the difference in PDLC response, osteoclast recruitment was faster in younger rats than in older rats when exposed to orthodontic force, suggesting greater reactivity in the younger periodontium.^23^ The integrity of lacunar-canalicular network and the mechanosensitivity of osteocytes, which play an important role in mechano-transduction and bone remodeling, gradually decrease with age.^43,44^ It is proved that, with aging, the number of osteocyte dendritic processes, which was important for mechanosensitivity and communication between cells, decreased gradually.^45^ The deterioration of the lacunar-canalicular network reduced the connection between osteocytes and between osteocytes and bone matrix, which influences the supplement of nutrition of osteocytes, reduces their mechanosensivity, and causes the lower bone responsiveness in the elderly.^41,45^

#### Reduced osteoclast activity

Osteoclasts were the first factors to be blamed for this phenomenon since OTM is closely related to bone resorption. Derived from peripheral blood monocytes, osteoclasts are regulated by both systemic signals (e.g., hormone levels) and local stimuli (e.g., mechanical loading) to maintain the homeostasis and adaptive properties of bone.^46^ Unlike the adolescent skeleton, which possesses great potential for bone growth and metamorphosis, the adult skeleton is relatively stable and inactive. At one extreme, almost 100% of the skeleton undergoes remodeling in infancy, whereas in adults, only 10% of the skeleton is renewed per year.^47^ Compared with adults, adolescents have higher circulating concentrations of bone turnover markers, reflecting their high growth velocity and rapid bone turnover.^48^ In humans, the increase in the levels of biological markers of bone turnover (e.g., osteocalcin, bone alkaline phosphatase (ALP), and C-terminal telopeptides of type I collagen (CTX-1)) stops at ages of 11 to 13 years and thereafter declines towards adult levels in late adolescence. Levels of these factors remain stable throughout adulthood, with a slight increase observed in postmenopausal women and older men.^49^ The rate of alveolar bone turnover follows a similar trend to the general skeletal system. Misawa et al.^50^ studied 110 male rats aged 6 weeks to 100 weeks and reported that the level of bone resorption decreased with advancing age, which may result in an age-related delay in OTM.^26^ Li et al.^51^ also reported that the magnitude of the increase in RANKL and tartrate-resistant acid phosphatase (TRAP)-positive osteoclasts in gingival fluid was greater in younger rats. These findings have been further supported in animal models of distinct osteoclast activities. Verna et al.^52^ applied orthodontic force to high-turnover, low-turnover, and control rats. The high-turnover group had the fastest OTM (Table 1).

**Table 1:** Characteristics of studies (using OTM model) about interventions in this review

Items		Author/year of publication	Study design	Sample size	Age	Intervention	Outcomes
Surgery-assisted OTM	Corticotomy	Abbas, 2016^167^	RCT (split mouth)	20 patients with Class II Division 1 malocclusion	15–25 years old	Corticotomy or piezocision	Corticotomy: 1.5 to 2 times faster piezocision: 1.5 times faster.
	Laser-assisted flapless corticotomy	Seifi M, 2012^93^	RCT (split mouth)	8 rabbits	–	Er,Cr;YSGG laser assisted flapless corticotomy	Laser: 1.7 times faster
	Piezocision	Charavet C, 2016^94^	RCT	24 patients with mild overcrowding	20–46 years old	Piezocision	Piezocision: 1.43 times faster
	Micro-osteoperforations (MOPs)	Alikhani M, 2013^95^	RCT	20 patients with Class II Division 1 malocclusion	Average 24.8 years old	MOP	MOPs: reduce duration by 62%.
	Periodontally accelerated/augmentation osteogenic orthodontics (PAOO)	Alsino HI, 2022^99^	Systematic review	8 RCT with 175 patients	18.8–29.6 years	PAOO	PAOO: 1.39–1.61 times faster and tends to increase the thickness of alveolar bone.
		Jing WD, 2020^100^	RCT	30 patients with Class III malocclusion	Average 22.6 years old	PAOO	PAOO group showed 0.649 mm more BT gain at labial sites and 0.740 mm less BT reduction at lingual sites and 0.473 mm more WKG gain.
		Ahn HW, 2016^101^	RCT	30 patients with Class III malocclusion	17–29 years old	PAOO	PAOO: 1.25 times faster without vertical alveolar bone loss.
		Ma ZG, 2016^102^	Case report	11 patients with Class II or III malocclusion	Older than 18 years	PAOO	The vertical bone height increased 3.63 mm after PAOO.
Physical stimulation	Vibration	Pavlin D, 2015^111^	RCT	45 patients with premolars extraction	12–40 years old	30H vibration for 20 min per day	Vibration: 1.5 times faster.
		Katchooi M, 2018^109^	RCT	26 patients with premolars extraction	15–45 years old	30 Hz vibration for 20 min per day	Vibration does not increase the rate of OTM.
		Woodhouse NR, 2015^110^	RCT	81 patients with premolars extraction	Average 14.1 years old	30 Hz vibration for 20 min per day	Vibration does not increase the rate of OTM
		Alikhani M, 2018^115^	RCT	206 SD rats	120 days old	different, frequency and duration	30 Hz: 1.45-fold faster; 60 Hz: 2.1-fold faster; 120 Hz: 2.4-fold faster;
		Sasaki K, 2022^112^	RCT (split mouth)	24 wistar rats	25 weeks old	with or without local injection of SB431542 (inhibitor of TGF-β) + vibration	The acceleration effect of vibration depends on the activation of NF-κB-TGF-β-RANKL axis in osteocytes.
		Alazzawi MMJ, 2018^121^	RCT (spilt mouth)	80 SD rats	6 weeks old	laser or LIPUS or combination of laser and LIPUS	Laser: 600 μm more; LIPUS: 300 μm more and combination of laser and LIPUS: 1000 μm more.
		Arai C, 2020^120^	RCT	26 Wistar rats	12 weeks old	LIPUS	LIPUS: 130 μm more.
		Wu T, 2024^122^	RCT	27 SD rats	6 weeks old	LIPUS	0.5-fold faster and higher bone density and lower vertical bone loss.
		Zhou J, 2023^123^	RCT	24 SD rats	6 weeks old	LIPUS	2-fold faster and higher bone density and thicker alveolar bone at pressure side.
		Xin, 2023^124^	RCT	36 SD rats	6–8 weeks old	LIPUS	1.5-fold faster and 1.3-fold more bone formation at the tension side.
	Electromagnetic fields	Shan Y, 2021^125^	RCT	105 Balb/c mice	–	SMF	SMF: 34.67% more.
		Wang Q, 2023^131^	RCT	16 SD rats	6 weeks and 16 weeks	Application of the device that could convert occlusal forces into alternating fields	Young rats: increased by 34%; Old rats: increased by 164%
	Low-level laser therapy (PBM)	Wang X, 2023^132^	Narrative review	–	–	PBM	PBM: shorten by nearly 26% to 40%
	Electricity current	Davidovitch Z, 1980^134^	RCT	9 cats	10–12 months	Micro-electricity	Micro-electricity activates neighboring PDL cells and enhances the cellular activities.
		Spadari GS, 2017^135^	RCT	32 Wistar rats	90 days	Micro-electricity	Microcurrent increased the number of vessels and osteoclasts and the level of tissue remodeling cytokines TGF-β1, VEGF, and bFGF
Agents and molecules	RANKL	Chang JH, 2020^164^	RCT	24 Wistar rats	15 weeks old	Injectable gel loading RANKL	1.3 times faster.
	PGE2	Brudvik P, 1991^162^	RCT (spilt mouth)	25 Wistar rats	–	Local injection of 0.1 mL of PGE2	Increases the risks of root resorption.
	EGF	Alves JB, 2009^136^	RCT (spilt mouth)	96 Holtzman rats (divided into four groups)	–	Local injection of EGF-liposome	About 300 μm more than control.
	Sclerostin	Lu W, 2019^137^	RCT (spilt mouth)	48 Wistar rats	6 weeks old	Local injection of sclerostin protein carried by hydrogel	About 200 μm more than control.
	Osteocalcin (OC)	Hashimoto F, 2001^138^	RCT	48 Wistar rats	5 weeks old	Local injection of OC	0.1 μg OC: 1.47 times faster; 1 μg OC: 1.52 times faster; 10 μg OC: 1.21 times faster.
	1,25-(OH)_2_D_3_	Takano-Yamamoto T, 1992^139^	RCT (spilt mouth)	60 Wistar rats	7 weeks old (*n* = 30); 28 weeks old (*n* = 30)	Local injection of 1,25-(OH)_2_D_3_	Young rats: 1.26 times faster; Old rats: 1.54 times faster.
	Teriparatide	Soma, S,1999^141^	RCT	48 Wistar rats	–	local injection of PTH	The injection of PTH: 2-fold faster without more alveolar bone loss
		Souza^142^	Systematic review	3 articles for final analysis	–	Drug concentration, administration and time for drug release	Major effectiveness: local injection of PTH (1–34) and PTH (1–38) and release in gel increased the time for drug release.
	Nitrous oxide	Sun Y, 2022^143^	RCT	24 C57B/6j	–	Local injection of L-arginine (NO precursor) group	The application of NO inhibitor decreases the amount of tooth movement.
	Combined bioactive factors in platelet-rich plasma	Navya S, 2022^140^	RCT (spilt mouth)	11 patients with Class I malocclusion	18–30 years old	Local injection of PRP or Vitamin D	PRR: 6.1 mm; Vitamin D: 4.1 mm.
	6-Shogaol	Zhu X, 2021^148^	RCT	18 SD rats	12 weeks old	Intraperitoneal injection of 6-shogao	Increased by 2-fold.
	4-Hexylresorcinol (4HR)	Choi KH, 2020^149^	RCT	30 ovariectomized SD rats	6 weeks old	Subcutaneous injection of 4HR	High-dose of 4HR: about 1 mm more
	Sema 3A	Satomi K, 2022^146^	RCT	48 C57BL/6j mice	7–8 weeks old	periodontitis + local injection of Sema 3A	In the OTM model with periodontitis, local injection of Sema3A inhibits alveolar bone loss, but does not inhibit tooth movement.
	Sir3	Li, 2023^45^	RCT	C57/BL mice	4 months and 20 months old	Running exercise	Running exercise significantly increased femur bone density in young mice but the density in old mice kept decreasing.

#### Decreased pro-inflammatory cytokines

Following the application of orthodontic force, the inflammatory response in the periodontium is activated.^53,54^ The level of local sterile inflammation plays a crucial role in osteoclast precursors recruitment and maturation, determining velocity of alveolar bone remodeling and OTM. Pro-inflammatory cytokines, including interleukin (IL)-1β, IL-6, IL-17, tumor necrosis factor alpha (TNF-α), and prostaglandin E2 (PGE2), etc., exacerbate and maintain the osteoclast-inducing inflammatory milieus during OTM,^55,56^ while other anti-inflammatory cytokines, including interferon beta (IFN-β), IL-4, IL-10, and C-C motif chemokine ligand 2 (CCL2), etc., quench overactive inflammatory response and bridge to following tissue reconstruction.^57^ Downregulation of pro-inflammatory cytokines in adults may partly explain the reason of OTM retardation. Iwasaki^20^ demonstrated that the rate of tooth movement was positively related to the ratio of IL-1β/interleukin-1 receptor antagonist in gingival crevicular fluid (GCF). It was found^58^ that PGE2 levels were significantly increased at 24 h after orthodontic force applied in both adolescents and adults, whereas IL-6 and granulocyte-macrophage colony-stimulating factor (GM-CSF) levels were significantly increased only in adolescents, suggesting that cytokine levels were more responsive in adolescents in the short term after orthodontic force application. Chibebe et al.^59^ reported that PGE2 concentrations in GCF of adolescent subjects ((13 ± 2.1) years old) increased significantly from baseline to 21 days and then decreased during the next week, while PGE2 levels of adults ((24 ± 2.1) years old) did not show significant changes. A systematic review^60^ summarized the changes in cytokine levels in GCF during orthodontic treatment. The result showed that younger patients (less than 16 years old) exhibited more pronounced cytokine levels and faster OTM rate than older patients (16–43 years old). These results imply a slower increase in osteoclastogenesis-related cytokines may hinder the maturation and recruitment of osteoclasts in older individuals.^23^

## Alveolar bone mass

Bone remodeling, a tightly coupled process consisting of bone resorption and formation, lasts throughout life. Under physiological conditions, bone catabolism and anabolism are in a dynamic equilibrium that maintains the bone mass and mechanical properties. However, with hormone disorders or aging, bone resorption may exceed bone formation, causing systemic bone loss, i.e., osteoporosis.^61^ Likewise, when orthodontic force is applied, both osteoclasts and osteoblasts are activated, resulting in increased alveolar bone metabolism. However, compared to adolescents, adults are more likely to suffer from noticeable alveolar bone loss posttreatment, even with the same treatment plan and force system are chosen, which manifests as a dimensional loss in alveolar bone and gingival recession, compromising periodontal health and smile aesthetics. Several studies based on 2D radiographs have shown that the distance from the cementoenamel junction (CEJ) to the alveolar crest increased more in adults after orthodontic treatment.^62–64^ However, some studies have not reported significant differences owing to deviations in the angle of the images and their deformation.^29,65^ Cone beam computed tomography (CBCT) is a more accurate method for measuring alveolar bone changes, both vertically and transversely. Qin et al.^66^ found that the lingual and labial distance from the CEJ to the alveolar crest of the mandibular incisors increased significantly after extraction treatment in adult patients, although it could recover to some extent during retention. After the correction of Class II malocclusion via extraction, Zheng et al.^2^ compared changes in parameters related to the alveolar bone around the central and lateral incisors between the two age groups. They found a significant reduction in alveolar bone thickness and a greater increase in the total distances from the CEJ to the alveolar crest in the adult group (18–35 years old), indicating greater alveolar bone loss in adults. Jing et al.^67^ included 54 adult patients with skeletal Class III malocclusion to perform multivariate analysis and they found that older age and the history of orthodontic treatment could significantly increase the prevalence of bone fenestration. Furthermore, Luo et al.^68^ included 499 orthodontic patients in a retrospective study to perform multivariate analysis of alveolar bone dehiscence and fenestration in anterior teeth after orthodontic treatment. They found that age (more than 18 years old) was one of the risk factors that significantly influenced the prevalence of anterior tooth dehiscence. In addition to measurements obtained directly from radiographs, clinical features provide a wealth of evidence. In adults, the incidence of open gingival embrasures was reported to be 22–38% for maxillary central incisors and 36% for mandibular central incisors.^69,70^ In contrast, Burke et al.^71^ suggested that more than 15% of all adolescent patients could be expected to exhibit “black triangles” after orthodontic treatment. Moreover, some studies have reported that correcting Class II malocclusion did not increase the incidence of gingival recession in adolescents.^72,73^ However, the occurrence of open gingival embrasure can also be related to other factors other than orthodontically induced alveolar bone resorption, such as dysmorphic and inadequate alveolar crest due to initial tooth crowding. Further well-designed CBCT-based studies are needed. Several factors, such as the sex and age distribution, growth patterns, types and degrees of malocclusion, distance of incisor retraction, orthodontic force system, and oral hygiene, etc., should be carefully controlled in future studies.

### Underlying mechanisms

As in other types of bone, macroscopic geometric changes in alveolar bone after orthodontic treatment are physiological adaptations to sustained active mechanical stimulation that mediates bone resorption and formation around the teeth according to the variable stress distribution of periodontal fibers and Wolff’s law. Hence, the inadvertent loss of alveolar bone after orthodontic treatment in adults can be attributed to both local and systemic causes that contribute to the disruption of alveolar bone homeostasis.

#### Age-related periodontium degeneration

In general, craniofacial bone growth progresses from superior to inferior, and the jaw grows downward and forward. To maintain functional occlusion, teeth continue to erupt and move. Therefore, tooth eruption contributes greatly to the vertical development of the dentoalveolar bone, a process that peaks at ~12 years of age in girls and 15 years of age in boys.^73,74^ A retrospective study based on lateral cephalograms of 142 males and 159 females revealed that vertical growth of the facial skeleton continues after puberty, but the amount of growth decreases steadily and appears to be clinically insignificant after the second decade of life^75^ and alveolar bone height continues to increase from 10 to 17 years of age.^76^ Ongoing growth and development of alveolar bone may partly explain the relatively undetected alveolar bone loss in adolescent patients. However, height and thickness of alveolar bone diminish during healthy aging.^27,77^ Bondevik^78^ compared two sets of cephalograms from 164 subjects taken ~11 years apart. It was found that the height of the mandibular alveolar process decreased by 0.80 mm in men. A 2-year longitudinal study also revealed that the rate of alveolar bone loss increased rapidly between the ages of 33 and 56.^79^ Therefore, the baseline disparity of periodontium between adolescents and adults may also contribute to this issue. PDL, the fundamental mediator of tooth eruption and alveolar bone development, plays a prominent role in maintenance of alveolar bone morphology and mass.^80^ However, PDL turnover is incredibly fast. Collagen fibrils within only have a half-life of several days, maintaining the tissue integrity of PDL. Lim et al.^38^ compared the structure of periodontium of 4, 10, 25, and 50-week-old mice and they found that the thickness of periodontal ligament, cell proliferation, the quantity and quality of collagen fibers decreased with age. Mechanistically, age-related impairment of collagen metabolism may result in functional loss of PDL, which contributes to periodontal degeneration in adults.

#### Reduced bone anabolism/imbalanced bone metabolism in adults

Bone mineral density peaks in the third decade of life and then gradually declines.^9^ Some studies have highlighted the contribution of elevated osteoclast activity. Aging may enhance the host response to the oral microbiota, resulting in the secretion of cytokines associated with bone resorption, which would help to create an environment that is more conducive to bone resorption.^81^ Aging promotes the expression of RANK on osteoclast precursors and RANKL on supporting mesenchymal stromal cells^82^ and promotes osteoclast function in healthy gingiva by upregulating the expression of BMP-3, MMP-8, and secreted phosphoprotein-1.^81^ Studies revealed that PDLCs from older subjects secreted markedly more PGE2 and IL-1β in response to compressive force than did those from younger subjects,^77,83^ which is consistent with the microenvironment being more conducive to bone resorption.

**Fig. 4:**
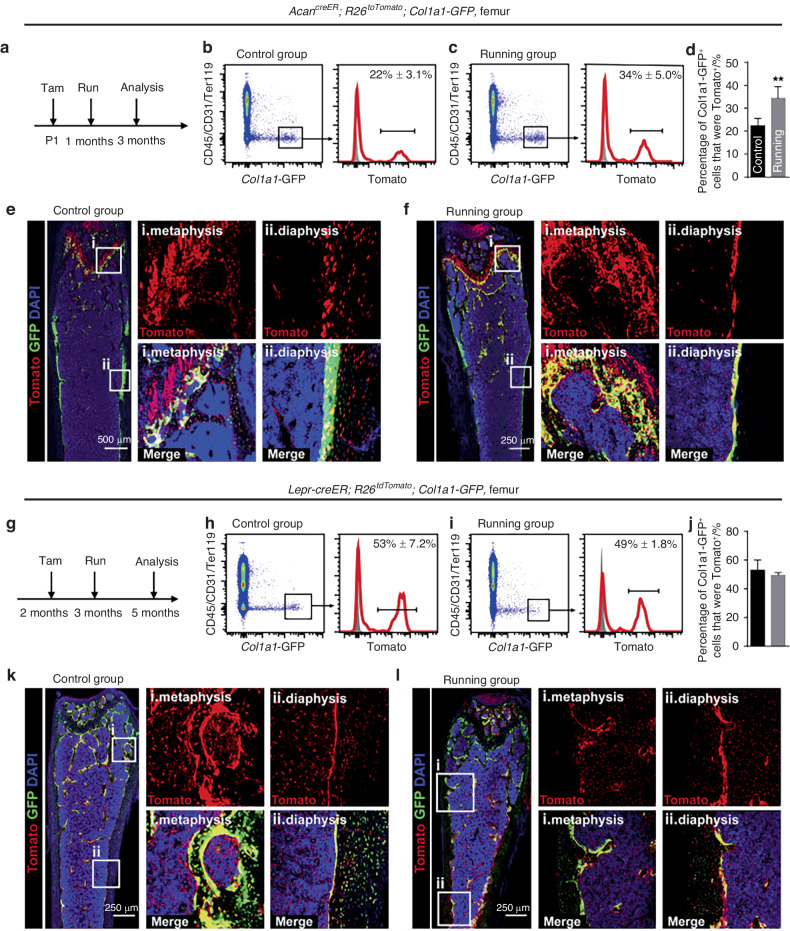
Mechanical stimulation enhances osteoblastogenesis by perinatal chondrocytes but not by adult skeletal progenitors. **a**–**f** show the results from 1-month-old mice, and **g**–**l** show the results from 3-month-old mice. Skeletal progenitors are marked by Tomato, and osteoblasts are marked by GFP. Mechanical stimulation (running) increased the number of Tomato+ osteoblasts in the GFP-labeled osteoblast pool in younger mice, while in older mice, the difference is not significant. Open Access^46^ (from Shu et al., *Cell Stem Cell* 2021, 28(12):2122–2136.e3)

However, as described in section "Reduced osteoclast activity" of this review, age-related functional changes in alveolar osteoclasts remain controversial. An anabolic reduction of alveolar bone may dominate this process. Likewise, although reduced osteoclast function was observed,^49^ catabolism still outweighs anabolism in age-related bone degeneration, which is defined as type II osteoporosis. In a histomorphometric study, Misawa et al.^50^ reported that the levels of bone formation rate (μm^3^/day) in rats decreased with increasing age from 6 to 100 weeks. Several age-related manifestations in the PDL also indicated a decrease in osteogenesis. The vascularization capacity^39^ and the number of active osteoblasts^4^ in the PDL showed age-related decreases. Wu et al.^6^ isolated human periodontal ligament stem cells (hPDLSCs) from different age groups (18–30, 31–45, and 46–62 years old). The results showed that the proliferation and osteogenic capabilities of PDLSCs decreased with age. Differences in bone metabolism may also be associated with the distinct types of bone-forming cells in adults and adolescents. Postnatal osteoblasts arise mainly from chondrocytes in adolescence and contribute to bone lengthening, while leptin receptor-positive (*Lepr*^+^) bone marrow-derived mesenchymal stem cells (BMSCs) dominate in adulthood and are responsible for adult osteogenesis in the steady state.^46^ Intriguingly, physical stimulation, such as running, could enhance osteogenesis by developing progenitors but not *Lepr*^+^ BMSCs (Fig. 4).^46^ Premature aging of skeletal progenitors could also explain several of these findings. Skeletal stem/progenitor cells (SSPCs) have been recognized as indispensable for adult skeletal remodeling.^46,84^ More recently, Yang et al.^85^ established an age-related bone loss model. Intriguingly, instead of chondrocytes, periosteal cells or osteoblasts, the premature aging of *Prx*^+^ SSPCs was shown to be responsible for attenuated bone mechanosensing, resulting in progressive bone loss. Given that bone cells and hematopoietic cells share the same harbor, cell interactions in the niche play a vital role in maintaining stemness and bone homeostasis.^86^ However, age-related functional changes in hematopoietic cells may result in imbalanced bone metabolism prior to noticeable changes in hematopoietic or immunological phenotypes. Megakaryocytes (MKs) can promote osteoblast proliferation and inhibit osteoclastogenesis through direct cell-to-cell contact.^87,88^ It was reported that aging could impair MK-stimulated osteoclast proliferation and cause bone loss (comparing mice aged 3–4 months, 11–14 months, and 22–24 months).^86^ Conversely, aging SSCs also provide an inflammatory and degenerative niche for blood lineage, which leads to hematopoietic senescence, therefore, influencing osteoclastogenesis. Notably, in addition to decreased bone- and cartilage-forming potential, Ambrosi et al.^89^ expounded that intrinsic aging of SSCs also produced stromal cells that express high levels of RANKL, CCL11, and CSF1, which created an inflammatory milieu for myeloid skewing and enhanced osteoclastic activity. The study revealed an exhilarating mechanism of bone integrity loss during skeletal aging. Although these results were obtained from long bones, they are still helpful in understanding age-related differences in orthodontic alveolar bone remodeling.

## Intervention methods

Various interventions have been proposed to meet the growing clinical need to accelerate OTM and reduce periodontal risk in adult orthodontic patients. The aforementioned evidence highlights the underlying mechanisms of this phenomenon, and therefore the core philosophy is to simultaneously promote bone resorption and formation in response to orthodontic force, thereby rejuvenating alveolar bone metabolism. In this section, these strategies are grouped into three categories according to their invasiveness and mechanisms. However, the paradoxical aim raises a tricky question: how to manage the adverse effects of catabolic activation during orthodontic treatment, e.g., excessive root resorption, and strike a new balance between bone resorption and bone formation.

### Surgery-assisted alveolar bone activation and augmentation

Since dense alveolar cortical bone could be the main source of resistance, in 1959, Kole^90^ proposed a pioneering periodontal surgery to accelerate OTM in adults by destroying continuity of cortical bone and moving the bony “block”. The procedure involved osteotomy of the cortical layer on both the buccal and lingual sides over the entire alveolar height. Active tooth movement was observed within 8–12 weeks, without significant root resorption.^90^ However, OTM acceleration after surgery is not as simple as lowering bone density or reducing resistance. As early as 1977, Epker and Fish reported an increase in bone remodeling in the operation area after surgery, and this phenomenon was subsequently reported as the regional acceleration phenomenon (RAP).^91^ However, accelerated OTM often occurs after orthognathic surgery, although osteotomy lines less involve alveolar bone. Now, RAP is commonly accepted to be driven by local hypoxia, reactive oxygen species (ROS) accumulation, etc., which creates osteoclastic bias in alveolar bone, thus contributing to the acceleration of OTM.^92^ Moreover, given that bone healing could be considered as postnatal bone development, which largely recapitulates phenomenological events and the expression patterns of development-related genes,^93^ microtrauma-mediated alveolar reactivation could also be understood as an effective approach that works via local bone rejuvenation. Recently, improved minimally invasive methods, including laser-assisted flapless corticotomy,^94^ piezocision,^95^ and micro-osteoperforations (MOPs),^96^ which are modifications of traditional corticotomy, have been proven to be equally effective. Generally, minimally invasive surgery could significantly accelerate tooth movement by 0.52 mm and 0.59 mm at 1 month and 2 months of follow-up, respectively.^97^ Despite efforts to reduce the invasiveness of surgery, there is currently insufficient evidence to suggest that a single use of MOPs could significantly shorten the overall treatment time. This is due to the local inflammation resolving, emasculating RAP over time.^97^ RAP generally peaked 1 to 2 months after surgery, typically lasted approximately 4 months, and gradually disappeared after 6 months.^98^

In addition to causing microtrauma, simultaneous bone grafting is conducted at the labial sites of anterior regions. Periodontal accelerated osteogenic orthodontics (PAOO)^99^ augments recipient beds of alveolar bone, enabling OTM beyond the original physiological boundaries as well as averting the possibility of additional alveolar resorption that may be caused by corticotomy. Compared to traditional orthodontic treatment, PAOO increased the rate of OTM by 39%–61%.^100^ Jing et al.^101^ also reported that PAOO patients gained an average of 0.649 mm in labial bone thickness after orthodontic treatment. It has also been documented that the alveolar bone height in the operative area exhibited an increase of approximately 4 mm,^102^ in contrast to a decrease of 3 mm in the control group.^103^ Bahammam^104^ compared changes in bone density among three groups of patients who underwent incisor retraction: Group 1 received corticotomy without a graft, Group 2 received corticotomy with a xenograft, and Group 3 received corticotomy with bioactive glass. Group 2 exhibited the smallest decrease in bone density (−29.82% vs. −14.43% vs. −24.04%) after treatment and the greatest recovery (+0.87% vs. +31.99% vs. +13.71%) after a 9-month follow-up. This demonstrated that the type of bone graft materials may play an important role in determining the prognoses of bone augmentation. Given the constraints imposed by the limitations of surgical scope and the duration of RAP, further endeavors, such as the modification of surgical procedures and the development of bioactive graft materials, should be undertaken to enhance the efficacy of surgical-assisted solutions. Meanwhile, the question of whether PAOO increases the incidence of root resorption remains controversial. Pitfalls emerged when the operation was performed in specific regions, such as lingual sites of the anterior teeth or areas with inadequate interradicular spaces.^105^ In addition, elevation of flap on both buccal and lingual sides might increase the risk of gingival recession after surgery.^106^ All of which are needed to be addressed in future researches in field of orthodontic-oriented periodontal surgery.

### Physical stimulation

Due to their high degree of invasiveness and constrained OTM acceleration, periodontal surgeries are not always the most preferred option for patients. Physical stimulation exerts a non-negligible influence on the activation of biological pathways, which in turn determine the behavior and fate of periodontium cells. This approach is considered another promising regimen for OTM acceleration. Mechanical vibration, electromagnetic fields, electric currents and low-level laser therapy, etc., have been reported as nonsurgical modalities for acceleration.

Mechanical cues exert a broad influence on cells. In vitro studies have shown that circumstantial reciprocal loading can stimulate both osteogenesis and osteoclastogenesis by activating the mechanically gated cation channel Piezo1.^107^ High-frequency (≈30 Hz) and low-magnitude (≈0.3 g) vibration (LMHFV) is recommended to improve the bone mineral density of postmenopausal women.^108^ LMHFV has also been reported to promote bone remodeling through Wnt/β-catenin signaling in animal models and was proposed to affect estrogen receptor signaling and cytoskeletal remodeling.^109^ Consequently, supplemental vibration has been implemented with the objective of facilitating alveolar bone remodeling. Nevertheless, the effectiveness of 30 Hz vibration on OTM acceleration has been the subject of debate.^110–112^ A relatively higher frequency (70–120 Hz) may be more reliable for OTM acceleration.^113–115^ However, no further acceleration was observed when the duration of treatment exceeded a certain frequency and duration.^116^ Sasaki et al.^113^ demonstrated that supplementary vibration (70 Hz) accelerated tooth movement through the activation of the NF-κB-TGF-β-RANKL axis in osteocytes, which was contingent upon the loading conditions. Alikhani^116^ demonstrated in rats that the application of 60 Hz and 120 Hz caused 2.1-fold and 2.4-fold increase, respectively, in the rate of OTM, while 30 Hz resulted in only a 1.45-fold increase. It’s noteworthy that bone metabolic effect of high-frequency vibrations may depend on PDL-bone crosstalk and local biomechanical milieus. High-frequency vibration (e.g., 120 Hz) accelerates osteoclastogenesis by increasing inflammation in the periodontium only as a supplement to continuous static orthodontic force,^113^ while 120 Hz vibration promotes alveolar bone healing after tooth extraction by inhibiting osteoclastogenesis and promoting osteogenesis.^117^ Low-intensity pulsed ultrasound (LIPUS) is a special form of vibration with a frequency exceeding 20 000 Hz. It has been proven to accelerate the rate of soft and hard tissue healing and has been approved by the U.S. Food and Drug Administration (FDA) as a physical therapy modality.^118^ LIPUS achieves greater penetration than lower-frequency vibration. It has been demonstrated that LIPUS could facilitate mandibular growth in baboons^119^ and the healing process during orthodontic-induced root resorption.^120^ The encouraging effects on alveolar bone and the PDL indicate that LIPUS could be a potential adjuvant therapy for orthodontic treatment. In rodent studies, Arai^121^ and Alazzawi^122^ applied 1.5 MHz pulses during OTM and reported significantly increased tooth movement and increased compensatory bone formation or osteoblastic activity. Recently, Wu et al.^123^ and Zhou et al.^124^ reported that in rat model of OTM, LIPUS treatment could accelerate the rate of OTM, increase alveolar bone density and decreased vertical bone absorption. These results showed that LIPUS can accelerate bone resorption and bone formation. Similar to LIPUS, Xin et al.^125^ reported that the application of low-frequency high-intensity ultrasound (44 W/cm^2^, 28 kHz) in the rat OTM model could increase OTM by 1.5-fold and increase the bone formation on the tensile side by 1.3-fold.

**Fig. 5:**
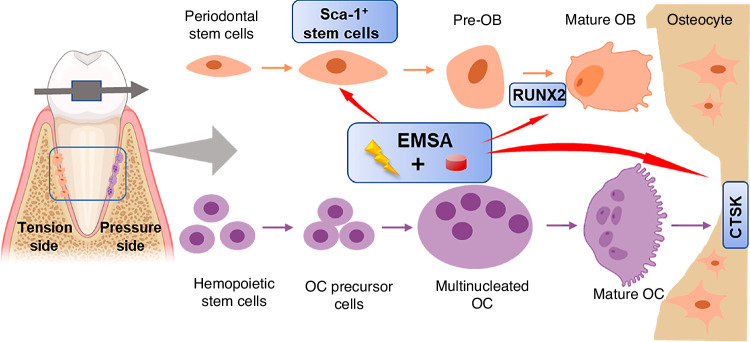
Mechanistic illustration of a novel occlusion-activated electromechanical dental aligner. With the synergistic effects of electromechanical intervention, aged rats demonstrated a 164% increase in OTM efficacy, which might be attributed to the simultaneous augmentation of osteoblast and osteoclast differentiation and maturation. Reprinted with permission^132^ (from *ACS Nano* 2023, 17:16757–16769). Copyright 2023, American Chemistry Society

As with mechanical vibration, both static^126^ and pulsed^127^ electromagnetic fields (EMFs) have been observed to accelerate tooth movement by increasing local blood flow and the osteoclastic activity. Moreover, pulsed EMFs are competent to promote osteogenesis.^128^ Non-ionized and non-thermal EMFs have been shown to induced biological effects in variety of tissues and cells, offering a contactless and atraumatic alternative in certain medical contexts. Researches have shown the anabolic and anti-inflammatory effects of pulsed EMF on bones and joints, and the FDA has also approved pulsed EMF as a treatment for bone nonunion. Intriguingly, akin to the aforementioned mechanical stimuli, the regulation of osteoblast or osteoclast behaviors may be distinct depending on the frequency and intensity of EMFs.^129^ Bhad et al.^130^ revealed that the application of pulsed EMF increased the rate of tooth movement by 31% in a clinical setting. Shan et al.^126^ reported that the application of a static magnetic field with a moderate flux density (20–204 mT) could also increase the rate of OTM by increasing the number of osteoclasts and accelerating the formation and clearance of the hyalinized zone. In a study published by Chen et al.,^128^ it was reported that intermittent exposure to low-frequency (16 Hz) pulsed EMFs promoted osteogenesis in vitro by activating Piezo 1-induced Ca^2+^ influx. In addition, it was shown that EMFs could influence the fluidity of cell membranes, which subsequently led to alterations in proliferation or differentiation signaling cascades.^131^ Notably, EMF devices could be integrated into orthodontic appliances. A recent study proposed a dental aligner that could convert occlusal forces into electric fields (0.6 V/mm), increasing the rate of OTM by 34% in young rats and 164% in aged rats (Fig. 5).^132^

As a highly concentrated electromagnetic wave, laser can also trigger beneficial response during OTM. Low-level laser therapy (LLLT), also known as photobiomodulation (PBM) therapy, has been employed in clinic for years. Accumulating evidence indicates that wavelengths ranging from 660 nm to 830 nm are the most commonly utilized wavelengths in accelerated orthodontics.^133^ These wavelengths have been shown to affect calcium ion channels and the morphology and function of the mitochondrial respiratory chain, resulting in increased calcium concentration and adenosine triphosphate (ATP) supply.

Bone, a natural piezoelectric biomaterial, is capable of generating electrical signals when exposed to mechanical stimuli, which is important for promoting bone growth and metabolism.^134^ As early as 1980, Davidovitch^135^ applied a 15 μA current in an experimental study of tooth movement in cats and found that electricity could significantly increase the rate of tooth movement. The current could activate the voltage-gated calcium channels on the cell membrane, which influences the intracellular calcium level thereby plays an important role in cell proliferation and differentiation.^134^ Moreover, Spadari et al.^136^ demonstrated that microcurrent application increased the number of vessels and osteoclasts and the levels of the tissue-remodeling cytokines TGF-β1, VEGF, and b-FGF. However, there’s still a paucity of high-quality evidence demonstrating the clinical efficacy of microcurrents in regulating alveolar bone remodeling. Relevant parameters, such as frequency, magnitude, and exposure duration, should be further identified.

### Agents and molecules

The intermittent use of additional stimulating devices may cause inconvenience, noncompliance, or undesirable side effects due to the presence of metal appliances or prosthetics. As previously discussed, since the levels of bone-forming and -resorbing cytokines and factors may decline in an age-dependent manner, the administration of molecules that are capable of regulating force-mediated bone metabolism may represent a promising strategy for the treatment of alveolar reactivation that should be considered. Multiple endogenic molecules directly or indirectly modulate bone metabolism. RANKL, PGE2, epidermal growth factor (EGF),^137^ sclerostin,^138^ and osteocalcin^139^ have been widely applied in regulating bone remodeling, including OTM. Vitamin D_3_ has shown encouraging results at the bench. Takano-Yamamoto et al. reported that the local injection of 10^−10^ mol/L 1,25-(OH)_2_D_3_ significantly increased the number of osteoclasts, accelerating OTM in young and mature rats by 126% and 245%, respectively.^140^ Platelet-rich plasma (PRP) contains a multitude of proteins and chemokines that facilitate bone healing and is also used for accelerating OTM, but the efficacy of PRP may rely on the concentration and delivery method.^141^ The administration of drugs at different frequencies may also result in distinct bone metabolic outcomes. For instance, the intermittent administration of PTH or its analogs may induce osteoblastogenesis, whereas continuous use may induce osteoclastogenesis.^142^ In their study, Souza et al.^143^ reported that the efficacy of teriparatide in accelerating OTM depended on the method of drug administration and the duration of drug release. They found that OTM could be accelerated by up to 3-fold. Endogenous gaseous molecules, such as nitric oxide (NO) and hydrogen sulfide (H_2_S), modulate a broad range of physiological processes, including bone metabolism. Sun et al.^144^ observed an elevated serum NO concentration and accelerated OTM after injection of the NO precursor L-arginine in a mouse model. The PI3K/Akt/β-catenin pathway was proven to be a downstream target of NO. Similarly, He et al.^145^ proposed that exploiting H_2_S donors may promote polarization of M1 macrophage, resulting in OTM acceleration via STAT1 signaling. Originally identified as a diffusible axonal chemorepellent that deeply involved in the development and function maintenance of nervous system, semaphorin3A (Sema3A) has recently garnered significant interests due to its dual effects on bone metabolism.^146^ The local injection of Sema3A could decrease alveolar bone loss in rats with healthy periodontium, without the inhibition of OTM.^147^ In addition, Sen et al.^148^ confirmed that the expression of Sema3A and its receptor in human periodontal ligament fibroblasts could be regulated by mechanical force. Specifically, they observed an increase in expression on the tension side and a decrease in expression on the pressure side, which could promote the expression of genes related to osteogenesis. Recently, Mei et al.^149^ revealed the multifunctional role of Sema3A in OTM, a key mediator of nerve-bone coupled adaptation towards mechanical loads, which induces bone formation through RhoA/ROCK2 pathway and following rearrangement of cytoskeleton and mitochondrial fusion.

It has been reported that natural compounds purified from herbs or plants can accelerate tooth movement, bringing promising prospects. 6-Shogaol, a compound derived from ginger with antioxidant activity, can promote the activation of JNK signaling and the expression of NFATc1,^150^ both of which play vital roles in the process of osteoclast formation and maturation. Simultaneous activation of bone resorption and formation may necessitate the use of formulas comprising of two or more molecules. Considering the potential interactions between candidates, molecules with dual effects have been suggested to be more desirable. In an experimental tooth movement model, the local injection of 4-hexylresorcinol (4-HR)^151^ simultaneously promoted the expression of BMP2 on the tensile side and RANKL on the compression side. This resulted in a significant acceleration of tooth movement and a slight decrease in the root-to-bone ratio (the ratio of the length of the root to the height of the alveolar bone) compared with those in the control group. The dual effects of 4-HR may be attributed to its ability to promote the expression of TGF-β, which is important in bone remodeling and may provide support when osteoclastogenesis is enhanced during the acceleration of OTM.

Notably, as reported by George et al.^152^ and Mohanakumar et al.^42^, levels of the cell senescence marker β-galactosidase decreased in both young and adult PDLCs that were activated. This indicated that anti-senescent or periodontium-rejuvenating treatments are needed for adult orthodontic patients. Nevertheless, few studies have concentrated on this prospective area of research. In the light of the broad scope of age-associated functional decline and disease, senolytics have been demonstrated to have a clear effect in alleviating senescent phenotypes and rejuvenating in various tissues and organs, including bone. Certain endogenous compounds were reported to rejuvenate aging BMSCs. Since the structure of lacuna-canalicular network may influence the mechanosensitivity of osteocytes, Li et al.^45^ demonstrated that the deletion of Sirt3, a longevity gene, could decrease the formation of dendritic processes in osteocytes, which could influence their mechanical transduction. The successful application of Sirt3 activator increased osteocyte processes and reduced the age-related bone loss in aged mice. In addition to its synthesis in the pineal gland, melatonin can be synthesized locally in bone. Emerging evidence indicates that the level of melatonin in the bone marrow declines with age, which may provide insights into the mechanism of senile osteoporosis to some degree.^153^ Xie et al.^154^ demonstrated that melatonin replenishment could promote the expression of the histone methyltransferase NSD2 in BMSCs isolated from patients with senile osteoporosis, increasing the accessibility of osteogenic genes and restoring the osteogenic potential of aged BMSCs. In recent years, an increasing number of molecules isolated from natural extracts have been confirmed to possess senolytic properties, indicating great therapeutic potential against age-related degeneration. Icariin, which is acquired from the Chinese herbal medicine *Herba Epimedii*, significantly rejuvenated BMSCs and macrophages in a senile osteoporosis-related inflammatory microenvironment by activating autophagy, which resulted in favorable effects on osteogenic and anti-inflammatory activity.^155^ Targeting ROS-induced bone senescence, ginkgolide B decreased the total ROS concentration in BMSCs from patients with senile osteoporosis and upregulated the ROS scavengers SOD2 and catalase, mitigating osteoclastogenesis and rejuvenating BMSCs.^156^

Utilizing extracellular vesicles (EVs) from mesenchymal stem cells, such as BMSCs and stem cells from human exfoliated deciduous teeth (SHEDs), is another promising strategy for bone-targeted rejuvenation therapy. These EVs can serve as carriers for biomolecules or act as a functional unit. Notably, MSC-derived EVs may have advantages in bone-targeted delivery or bone rejuvenation owing to their homologous targeting effect.^157^ Zhang et al.^158^ reported that EVs released from human BMSCs could deliver miRNA-935 to osteoblasts, silencing STAT1 to promote aged osteoblast proliferation and differentiation. Similarly, EVs derived from human BMSCs transmitted miRNA-186 to promote osteogenesis in osteoporotic rats through the Hippo signaling pathway, which also plays an important role in mechanical signal transduction.^159^ The systemic administration of SHED-EVs could target BMSCs, enhance the expression of telomerase reverse transcriptase and regulate the activity of telomerase to rejuvenate BMSCs and promote osteogenesis.^160^ Huang et al.^161^ reported that PDLSC exosomes promoted osteoclast differentiation by activating the extracellular regulated kinase (ERK) pathway and that systemic injection of exosomes increased the extent of OTM in mice. In addition,^162^ blocking human PDLSC-derived exosomes decreased the distance of OTM and decreased osteoclastic activity. Lei et al.^163^ reported that EVs from neonatal human umbilical cord MSCs (UCs) could transfer various anti-aging signals, such as proliferating cell nuclear antigen (PCNA), to senescent BMSCs. The delivery of these signals helps to alleviate senescent phenotypes, as indicated by enhanced self-renewal capacity and telomere lengthening, which thus reduced bone loss in aged mice.

Prior to commencing further clinical translational studies, several issues should be addressed. Given the pharmacokinetics and bioavailability of these agents, supraphysiological doses are often applied, which could lead to detrimental outcomes. For instance, intermittent injection of 0.1 μg/μl PGEs at 2-day intervals into the gingiva led to a greater risk of root resorption during orthodontic treatment.^164^ Patients who receive PTH might be at an increased risk of osteosarcoma.^165^ In addition to the potential side effects and the questionable stability of these agents after treatment, the administration methods should also be considered. In adult orthodontics, it is preferable that the metabolic modulation of bone is performed within the alveolar bone. Due to the conflict between the relatively lengthy duration of orthodontic treatment and the short half-life of free drugs, it is important to pursue methods of achieving sustained or on-demand delivery of bioactive agents. This could facilitate usage and potentially enhance clinical effectiveness. Hydrogels are commonly used as drug carriers because of their biomimetic physicochemical properties and capacity to create isolated internal environments. In recent years, there has been a growing body of research attempting to utilize hydrogels to deliver bioactive agents locally and sustainably in order to accelerate and augment orthodontic alveolar bone remodeling. Chang et al.^166^ and Lu et al.^167^ used a hydrogel system to deliver RANKL and PTH/PTHrP, respectively, via sustained release over a period of 30 days. Both of these hydrogels were used as fillers following treatment with MOPs and significantly increased the rate of tooth movement in rats. Recently, Jiao et al.^168^ synthesized an injectable biocompatible gelatin-reduced graphene oxide (GOG), which promoted osteoclastogenesis and vascularization and subsequently accelerated tooth movement when it was injected locally. However, the long-term effects of these agent carriers still require validation.

## Conclusion

For decades, clinicians and researchers have been obsessed with the differences in alveolar response between adults and adolescents in orthodontic treatment. Myriad attempts have been made to elucidate the discrepancies, while a lack of high-quality studies still plagued the field. By summarizing the current knowledge on the underlying mechanisms, the present review suggests that growth potential, age-related alterations in tissue microstructures, and cellular mechanosensing and reactions may be involved. These findings remind us of the intricate and profound age-associated changes in force-mediated alveolar bone metabolism and homeostasis maintenance, which still require further exploration. To accelerate the rate of bone turnover and augment bone mass, alveolar reactivation via surgery, noninvasive physical stimuli or bioactive agents is of great interest. However, as a caveat, it should be emphasized that balancing the levels of bone resorption and formation, mitigating side effects and achieving a win-win situation remain challenging. In light of the aforementioned evidence, the periodontium in adults was found to be pre-senescent. One can envisage the valuable prospects of using anti-aging molecules or senolytics administered via sustained drug delivery for periodontium rejuvenation. With the ever-increasing understanding of age-related bone biology, it is greatly anticipated that future inspiring works can benefit both the bench and the clinic, leading our way out of this “mystery forest”.
